# Association between Anxiety Status and Hydration Status in Spanish University Students

**DOI:** 10.3390/nu16010118

**Published:** 2023-12-29

**Authors:** María José Castro-Alija, Irene Albertos, Claudia Pérez Íñigo, María López, José María Jiménez, María José Cao, Megan Trayling, Jaime Ruiz-Tovar

**Affiliations:** 1 Recognized Research Group: Assessment and Multidisciplinary Intervention in Health Care and Sustainable Lifestyles, University of Valladolid, 47003 Valladolid, Spain; mariajose.castro@uva.es (M.J.C.-A.); claudia.perez.inigo@estudiantes.uva.es (C.P.Í.); maria.lopez.vallecillo@uva.es (M.L.); jose.maria.jimenez@uva.es (J.M.J.); mjcao@uva.es (M.J.C.); 2 Department of Biomedicin, Universidad Alfonso X, 28691 Madrid, Spain; megan.trayling@hotmail.com; 3 EUEF San Juan de Dios, Universidad Pontificia de Comillas, 28036 Madrid, Spain; jruiztovar@gmail.com

**Keywords:** anxiety, hydration, students, intake, Spanish

## Abstract

Anxiety disorders are a very common psychiatric pathology among young university students, and the strategies for its mitigation are limited to the consumption of anxiolytic substances. Adequate hydration is essential for staying healthy, as water is the main component of the human body and of several physiological processes. A state of dehydration, in addition to a negative water balance, has serious consequences on health status. We aimed to determine the association between the degree of anxiety and the level of hydration in Spanish university students. A cross-sectional, observational research study with a sample of 65 female university students was conducted. Over 90% of the study population shows anxiety levels above the 95th percentile. The individuals with anxiety above the 95th percentile showed a negative water balance. The consumption of coffee and herbal teas shows correlations with state anxiety and trait anxiety.

## 1. Introduction

Anxiety disorders are one of the most frequent psychiatric pathologies in developed countries and constitute an important public health problem. Several studies have associated this high prevalence of anxiety with lifestyle habits, work overload, low physical activity or difficulties in social relations, among others [1,2]. People often experience indirect manifestations of anxiety, such as waking up tired, having nightmares or feeling overwhelmed, internalizing this type of behaviour as something “normal” [3]. The state of anxiety leads to a worsening in the perception of life and increases the risk of cardiovascular, respiratory, digestive diseases and autoimmune issues [4].

The university community is subject to great work pressures, both physical and emotional, arising from financial dependence on their families, fear of failure or uncertainty about their future career. A recently published study, conducted at a Spanish university, showed that 11% of the students surveyed in different degree programmes within the Faculty of Health Sciences regularly consumed benzodiazepines. Although the anxiety levels in this university community were not specifically studied, stress levels were analysed and considered to be medium-high in 64.5% of men and 85.7% of women. As a result, 29.2% of students considered their state of health to be fair, bad or very bad [5]. These data are in line with other studies published in university communities in Spain, who have identified the impact of factors such as age, gender, self-esteem, sleep quality and living conditions of university students on their anxiety levels.

Other aspects to analyse were specific behaviours related to alcohol consumption, tobacco, and Internet use, which demonstrated a strong association with psychological distress in the population of university students [6,7].

The knowledge of these data should alert both the university community and society in general to initiate prevention strategies that allow for an exhaustive control of people at risk, make an early diagnosis of the appearance of symptoms and establish measures to alleviate the consequences of this. These consequences can vary from simple failure in studies to cases of suicide that have been described in the literature [8].

There are different methods for early diagnosis of anxiety states. One of the most frequently used tools is the State-Trait Anxiety Inventory (STAI) questionnaire. This questionnaire, which has been validated in different languages [9], differentiates between two situations: state anxiety and trait anxiety. This psychometric questionnaire contains 40 items, half of them belonging to the state subscale, which is made up of phrases that describe how the respondent feels at the time of the interview. The other half belongs to the trait subscale, which includes phrases that describe how the respondent usually feels. The former defines stressful situations that a person faces at a certain time in his or her life, while the latter refers more to the person’s own behaviour under normal conditions. Although state anxiety is more susceptible to change in specific situations, there is often a correlation between the two, as people with anxious personality traits are also more likely to experience changes in acute situations [9].

Adequate hydration is essential for mental and physical performance and overall health. Water is the main component of the human body and is essential for life and health. It is crucial for the proper functioning of several physiological processes, so inadequate hydration status is associated with poor health [10,11]. There are numerous factors, such as climate, physical activity or weather, among others, that influence water needs, even modifying them [10]. When the total body water is insufficient for the proper functioning of cells, organs and systems, it is called dehydration [12]. It is well known that severe dehydration is associated with health problems such as confusion, delirium and impaired immune, renal and gastrointestinal function. But even mild dehydration states have negative health consequences: headache, irritability, decreased physical performance and reduced cognitive function, among others [13,14,15,16,17,18]. It is known that inadequate water consumption has an impact on physical performance, which is why there are more studies that focus their lines of research on establishing prehydration strategies. A state of hypohydration can also have negative consequences, reducing levels of cognitive performance. This suggests that the state of hydration in students could have implications for their academic performance, in addition to their general health [12,14,15]. Recently published data suggest that the hydration status of the population is suboptimal compared to baseline values in up to 80% of individuals [19].

Water is understood as an essential nutrient for life that allows the development of different bodily functions, since it acts as a carrier, solvent, thermoregulator and reagent. Its daily elimination through urine and faeces, perspiration, and breathing, requires adequate replacement through intake to maintain optimal hydration. Its involvement in the transport of food is another issue to analyse, due to its relationship with food absorption. In this way, a meal with a deficient water intake produces a sensation of heaviness that may even be painful, while if it is accompanied by a high intake, it can produce a sensation of excessive fullness. The body’s fluid level is determined by the water balance, defined as the balance between water input and output. It is a dynamic process, influenced by various environmental conditions, availability of food and drink, physical activity, thirst and hormonal mechanisms. It is precisely regulated over a 24 h period, as intake and losses must be equal so that, under warm ambient temperature conditions and moderate activity levels, it remains relatively constant [10,12]. Several biomarkers of hydration status have been proposed, such as urine density, colour or osmolality. Recently, a hydration status questionnaire (HSQ) has been developed and validated using different biomarkers in an adult population. The HSQ provides information on hydration status, water elimination and intake, as well as water balance, and could be applied as a screening method to detect individuals at risk of dehydration [20].

A recently published study on aeronautical military personnel in Spain has shown an association between insufficient hydration status and the degree of anxiety. This finding has been related to an increased degree of pathological alertness or restlessness among the dehydrated personnel. A possible catecholaminergic response to the state of dehydration has been hypothesised, which may derive from a possible inflammatory response, as dehydration is identified as an aggression to the organism. These findings denote the importance of establishing an adequate hydration plan due to its implications on physical and mental performance [21].

The research hypothesis of this study was that a negative water balance is associated with increased anxiety levels. To this end, the following objectives were set: The primary objective was to associate the degree of anxiety of university students with their level of hydration. Secondary objectives were to determine the degree of anxiety of these students and to analyse the students’ hydration status.

## 2. Materials and Methods

### 2.1. Study Population and Sample Size

An observational cross-sectional study was conducted in an initial sample of female undergraduate students pursuing degrees in Nursing and Biomedicine at the University of Valladolid (Spain) and the University Alfonso X of Madrid (Spain), during the 2022/2023 academic year. Non-probabilistic convenience sampling was used in this investigation. The sample size was calculated with a confidence level of 95%, an estimation error of 5% and an additional 10% overestimation to compensate for the lack of interest in taking part in the study. From the initial sample selected, undergraduate students who did not present the informed consent and/or submitted an incomplete questionnaire were excluded from the study. A minimum sample size of 50 participants was calculated in order to obtain clinically relevant results, without setting minimum limits per university degree or per academic year. 

The exclusion criteria were male gender (in order to have a more homogeneous sample, given that in both degrees the number of male students is very low), individuals with previous renal pathology, individuals presenting an acute inflammatory condition of any kind at the time of data collection, recent hospital admission for any cause, recent destabilisation of an underlying psychiatric condition and intake, during the week prior to inclusion in the study, of diuretics, steroidal anti-inflammatory drugs or non-steroidal anti-inflammatory drugs (NSAIDs).

### 2.2. Methodology

Data were collected through surveys that were passed digitally to university students and included individuals who voluntarily agreed to participate. 

### 2.3. Questionnaires Used

The degrees of state anxiety and trait anxiety were assessed through the STAI questionnaire, validated for the Spanish population [22]. The questionnaire is divided into 2 groups of questions: some aim at determining state anxiety and others at assessing risk anxiety. Each question is assigned a score and, at the end of the questionnaire, the points obtained in each question are added together. Depending on the result of this sum, individuals are classified with a percentile whose values are previously established for the general population, differentiating between men and women, adolescents and adults.

Hydration status was determined using the HSQ, validated for the Spanish population by the Nutrition and Food Science research group at CEU San Pablo University [21]. HSQ includes the following: (a) personal information; (b) medical history; (c) hydration habits and knowledge; (d) a water, beverages and food frequency questionnaire; and (e) water elimination information. To estimate water output, three elimination pathways were taken into account: skin, kidneys and the digestive system. Urination and defecation were recorded on the basis of frequency, and to calculate sweating, a 10-point scale was used for both physical activity and sedentary conditions. This information allowed for the assessment of the estimation of water balance. This questionnaire determines fluid intake through the volume drunk, as well as through the water contained in the food ingested, which is estimated through food composition tables [21,23]. 

### 2.4. Statistical Analysis

The quantitative variables were defined using the mean and standard deviation, while the qualitative variables were characterised by the number and percentage of cases. Correlations between variables were performed using Student’s *t*-test for independent data, Chi-square and Pearson’s correlation test. Values of *p* < 0.05 were considered statistically significant. Statistical analyses were performed with the Statistical Package for the Social Sciences (SPSS, IBM, Armonk, NY, USA), version 28.0.

## 3. Results

A total of 65 subjects agreed to participate in the study and completed the questionnaires, 49 belonging to the Nursing Degree (75.4%) and 16 to the Biomedicine Degree (24.6%). Of all the students eligible to participate, the acceptance rate was 9.61% in the Nursing Degree and 8.9% in the Biomedicine Degree. The mean age of the participants was 20.4 ± 4.4 years. A distinction was also made according to the university year to which they belonged. There was a predominance of participation by students in the 4th year, comprising 60%, followed by those in the 3rd year, accounting for 23.1%. First-year students contributed 10.8% and second-year students 6.2%.

Among the personal history of the participants, the following stand out: anxiety (1.5%), unspecified musculoskeletal disorders (1.5%), asthma (2.5%), type 1 diabetes mellitus (1.5%), inflammatory bowel disease (1.5%) and hypothyroidism (2.5%).

### 3.1. Hydration Status

Only 2 participants (3.1%) reported motor or neurosensory difficulties that hinder fluid intake. Fluid intake habits are shown in Table 1.

In addition to water consumption, questions were asked about the preference to drink other beverages when thirsty. The responses show a preference for water at 92.3%, while 4.5% prefer soft drinks and 1.5% opt for natural juices or other types of beverages (1.5%). Isotonic or energy drinks are consumed by 4.6% of the students during physical exercise. 

The volume of the different types of beverages consumed by students is described in Table 2. The total volume of liquid ingested is 85.2 ± 32.9 L/month, i.e., 2.8 ± 1.1 L/day. The liquid they drink most is water (49.1 ± 26.5 L/month), followed by milk (7.9 ± 6.8 L/month).

The volume of fluid eliminated through urine, faeces and sweat was calculated (Table 3). Most of the volume of fluids eliminated is through urine and sweat, the latter including both sweat at rest and sweat during physical exercise (Table 4).

Water balance:

With the calculation of the volume ingested and eliminated, we established the water balance, which was negative, obtaining a mean loss of −10.1 ± 33.1 L per month, i.e., −336.6 ± 1104.4 mL per day.

### 3.2. State of Anxiety

It is striking that practically 90% of the studied population showed state anxiety levels above the 90th percentile and 46% above the 95th percentile (Table 5). The percentile values correspond to the data established for anxiety levels in the general population [9].

Regarding trait anxiety, 97% of the studied population showed levels above the 85th percentile and 44.6% levels above the 95th percentile (Table 5).

### 3.3. Correlations between Hydration and Anxiety Values

After evaluating the results obtained in both surveys, it can be observed that water balance is significantly lower in those individuals with state anxiety values above the 95th percentile (26.9 L/month in *p* < 95 vs. −13 L/month in *p* > 95; mean difference 39.9 L/month; 95%CI (4.5–84.5); *p* = 0.047). 

Coffee intake is significantly higher in those individuals with state anxiety values above the 95th percentile (0.7 L/month in *p* < 95 vs. 5.1 L/month in *p* > 95; mean difference 4.4 L/month; 95%CI (2.9–5.8); *p* < 0.001).

Herbal tea intake is significantly lower in those individuals with trait anxiety values above the 95th percentile (3.2 L/month in *p* < 95 vs. 0.7 L/month in *p* > 95; mean difference 2.5 L/month; 95%CI (0.5–4.5); *p* = 0.016).

## 4. Discussion

The results of this study show a negative water balance on average, revealing an insufficient fluid intake in the university community, which would be a population representation of adolescents and young adults. This reflects either a lack of awareness of the need to drink plenty of fluids or insufficient knowledge of the subject, which is less likely given that these are students in health science-related degrees. This may lead us to believe that outside the health sciences field, intake may be even lower. The average intake of 2.8 L of fluid per day may seem sufficient a priori, but based on the eliminations described and the physical activity they report, it is clearly insufficient, with an average deficit of 336.6 mL/day.

It has been reported in literature that the percentage of the general population with inadequate water intake ranges from 5 to 35% among European countries [24]. In Spain, the ANIBES study, conducted in 2013 [25], revealed that individuals had inadequate levels of total water intake when compared with the European Food Safety Authority reference values (2.0 L/d for females, and 2.5 L/day for males) [26]. In contrast, the recommendations established by the World Health Organization (WHO) advocate for a water intake of 3.7 L/day [27]. The recommendations of the National Health and Medical Research Council propose a water intake of 3.4 L/day for males [28], while the Institute of Medicine of the United States of America (IOM) suggests 3.7 L/d for males [29]. We must take into consideration that differences in water intake are not only a reflection of dietary habits, lifestyle choices, and environmental conditions, but also differences in the selection of methods used to evaluate total water intake [30]. This highlights the importance of evaluating water intake within the population, in our case, university students. Consequently, recommendations also depend on the methods used for quantification of the intake, taking into account all sources, both in foods and in beverages.

A recent study evaluating hydration status in aeronautical military personnel in Spain indicated that the total water intake was adequate for the majority of the population studied, with a mean total water intake of 3508 mL/day. This volume is significantly higher than the mean water intake of the participants in the present study. However, we must take into consideration that our study was conducted on females, while the study on military personnel was conducted on males. Furthermore, military personnel will have higher levels of physical activity than university students, and water losses through perspiration will also be significantly greater [21].

In our opinion, the key to an insufficient water intake may lie in the fact that only 53.8% of the participants recognize that they should ingest fluids before they feel thirsty. Water consumption is influenced by the different eating habits of students, in addition to the availability and access they have to it. In our case, they have public drinking water fountains that allow them to refill their bottles to take to classes. Even so, we consider it is important to develop population education programs to raise awareness of the need for adequate fluid intake, beyond just quenching the sensation of thirst.

This deficiency in fluids intake determines that the elimination of waste substances through urine is carried out in a habitual way, forcing renal function and increasing water reabsorption in the loop of Henle. This work overload, in the long term, may eventually lead to a deterioration of renal function and an increased risk of developing renal failure [31].

On the other hand, it is very striking that both state anxiety and trait anxiety levels are at such high levels. This reflects the degree of psychological pressure to which students are subjected, not only in relation to exams, but probably also to external social pressures, fear of failure, financial dependence or even future job uncertainty. This is the point on which all education professionals should take greater and earlier action, by reducing the unnecessary workload without reducing the degree of learning, eliminating the social stigma of failure and increasing the number of grants and funding for studies, ensuring that they are not only associated with academic records, but with other merits (research, teaching, innovation and so on). Finally, a social analysis should be carried out to evaluate the professional opportunities for university graduates. If it is not possible to guarantee them a decent job, it is important not only to consider increasing the number of university places but also to consider reducing them if necessary [5].

Students with higher levels of anxiety are found to have more negative mean water balances. This is consistent with the results obtained from the study conducted at the Gatorade Sports Science Institute, in which hypohydration appears to increase mental workload and lead to headaches, difficulty concentrating and increased fatigue. These results suggest that cellular hydration may affect neuronal function [32]. This raises the need to relate these data to the acquisition of skills during the training period and develop strategies that address the impact of mental health and hydration on academic performance and the results obtained in the affected group of students. Carretero-Krug et al. [21] described in their study on military personnel that the subjects that did not meet the hydration criteria had significantly higher scores in the STAI-state than the subjects that met the hydration criteria, reflecting a potential relationship between these two variables. In other studies, the relationship between hypohydration status and degraded mood was consistent. The findings showed that low water intake or low 24 h urine volume is associated with negative health outcomes, including an increased risk of chronic kidney disease, the development of kidney stones, and altered glucose homeostasis [33,34]. In additional studies, measurements of self-reported changes in mental state consistently found associations between dehydration and mood in conjunction with changes in performance. These findings also show the need to establish lines of research that address issues such as the impact of hydration on people’s mental state, body composition and its relationship with healthy lifestyles [35,36]. Neave et al. [37] tested young adults using a range of cognitive tasks, including attention and working memory, and showed that mood ratings significantly changed when individuals were given water. Individuals reported feeling more “calm” and “alert” immediately after water consumption. These results are in line with those of other young adult studies that found similar reports of “alertness” after water consumption [38].

Coffee intake is associated with increased anxiety-state. Caffeine is a central nervous system stimulant, which stimulates the release of catecholamines, causing an increase in blood pressure, respiratory rate and heart rate. Chronic caffeine consumption has been shown to produce nervousness, insomnia and restlessness, clinical signs very similar to those that occur in anxiety disorders. When coffee and anxiety are combined, all these symptoms are multiplied [39]. The pressure that competency evaluation periods can pose among students has an influence on their anxiety levels. If an increase in caffeine consumption is added to this, its impact and its relationship with the hydration of this population group can be studied.

In contrast, there are herbal teas, the intake of which is associated with less anxiety-trauma. Some of them, such as lime blossom, hypericum or valerian, have a sedative and relaxing effect on the central nervous system. Their consumption can reduce the degree of anxiety in people with anxiety-prone personalities [40].

### Limitations

First of all, it must be mentioned that the research was conducted only in the female population, given the small proportion of male students in these degrees. This was performed to homogenize the sample, but the results obtained cannot be extrapolated to males or even to the general population. Future studies must also include a male population.

After analysing the results, the low participation rate in this study is striking. The limiting factor was the excessively long hydration questionnaire. This assessment was indicated to us by many respondents who filled in both questionnaires. Therefore, new validated questionnaires should be designed, which allow an assessment of hydration status, but are also tools that are easier to apply in practice.

Although the hydration test has been validated in the Spanish population, it has certain limitations that should be taken into consideration. Firstly, the volume of water ingested through food, despite being estimated through food composition tables [23], is a very crude calculation, with great heterogeneity, even depending on the season of the year in which the questionnaire is completed. Some studies estimate that water intake through food is around 20% of the total volume ingested [20], which is higher than what we estimate in our study.

On the other hand, the volume of elimination is estimated from losses in urine, faeces and sweat. Within sweat losses, they differentiate between those eliminated during physical exercise, and even divide the type of exercise according to its intensity into light, moderate and heavy. However, the test does not define these exercise magnitude criteria, so there is a large inter-individual variability in the estimation.

Finally, the elimination volume does not take into account the loss of water vapour through respiration, which some studies estimate at almost one litre per day [24]. Therefore, the results obtained through the hydration test to establish water balance can possibly serve as a guide, but they should be interpreted with caution due to the aforementioned inaccuracies.

There are now applications on mobile phones and other electronic devices that allow real measurements of the amount of liquid ingested. This would probably allow a more accurate determination of water intake and even elimination. However, we opted for the HSQ method of assessment, as this allowed us to correlate the hydration parameters with the results obtained using the STAI questionnaire, while maintaining the anonymity of the data. However, it would be advisable to develop, in the future, a mobile application that could allow us to assess the actual intake and even make an approximation of the elimination, correlating it with the anxiety status.

## 5. Conclusions

Ninety per cent of the study population shows levels of state anxiety above the 90th percentile and 46% above the 95th percentile. In terms of trait anxiety, 97% of the study population shows levels above the 85th percentile and 44.6% above the 95th percentile. These findings highlight the need to identify the reasons that increase the level of anxiety in students in order to establish specific strategies that help them cope with it.

The mean water balance in the study population is negative, −10.1 L per month or −336.6 mL/day. Water balance is significantly lower in those individuals with state anxiety values above the 95th percentile. These results reflect the importance of health education in relevant aspects such as hydration and its impact on health, daily intake recommendations, influence of lifestyle habits on water consumption and its influence on physical and mental health. Coffee intake is significantly higher in individuals with state anxiety values above the 95th percentile, while herbal tea intake is significantly lower in those with trait anxiety values above the 95th percentile. High levels of coffee consumption are related to the appearance of symptoms such as anxiety, agitation, insomnia and gastrointestinal disorders, so it could be a risk factor in the appearance of mental problems. If, as in our case, we are starting with students experiencing high levels of anxiety, the habitual consumption of caffeine, due to its stimulating effects, can have negative consequences on daily coping with classes during the training period. This is linked to both anxiety and maintaining adequate hydration needed to achieve correct academic performance.

Based on these results, it would be important to recommend an increase in fluid intake as part of their treatment for individuals with anxiety or at risk of developing it, as well as to advise them to limit their coffee intake and/or replace it with herbal teas with a relaxing effect. However, further studies with larger sample sizes and direct measurements of both fluid intake and fluid elimination are needed to confirm these results.

## Figures and Tables

**Table 1:** Fluid intake habits.

	*n*	%
Carry a bottle of water when they go out	20	30.8
Carry a bottle of water when they go to university	49	75.4
Find it pleasant to drink water	60	92.3
Consume water outside of meals while at home	56	86.2
Drink water when exercising	62	95.4
Drink fluids before they feel thirsty	35	53.8
Feeling that drinking fluids makes them feel full	38	58.5

**Table 2:** Volume of liquids ingested, including water contained in foods.

Beverage	L/Month
Water	49.1 ± 26.5
Juices	4.1 ± 8.4
Soft drinks	1.6 ± 1.9
Milk	7.9 ± 6.8
Milkshakes and yoghurts	3.3 ± 5.1
Coffee	5.2 ± 5.5
Herbal teas	2.1 ± 4.2
Alcohol (Wine)	1.8 ± 2.5
Alcohol (Beer)	2 ± 3.1
Alcohol (Spirits)	1.8 ± 2.3
Water included in food	6 ±3.8
Total Volume Ingested	85.2 ± 32.9

**Table 3:** Volume of liquids eliminated.

	L/Month
Urine	44.7 ± 9.5
Faeces	4.2 ± 0.5
Sweat	46.5 ± 6.4
Total volume of fluids eliminated	95.3 ± 12

**Table 4:** Distribution of anxiety-state by percentiles.

	*n*	%
<P85	2	3.1
P85–P90	8	12.3
P90–P95	25	38.5
P95–P99	24	36.9
>P99	6	9.2
Total	65	100

**Table 5:** Distribution of trait anxiety by percentiles.

	*n*	%
P75–P85	2	3.1
P85–P95	34	52.3
P95–P99	24	36.9
>P99	5	7.7
Total	65	100

## Data Availability

The data presented in this study are available on request from the corresponding author. The data are not publicly available due to piracy.
